# BIM-Mediated AKT Phosphorylation Is a Key Modulator of Arsenic Trioxide-Induced Apoptosis in Cisplatin-Sensitive and -Resistant Ovarian Cancer Cells

**DOI:** 10.1371/journal.pone.0020586

**Published:** 2011-05-31

**Authors:** Zhu Yuan, Fang Wang, Zhiwei Zhao, Xinyu Zhao, Ji Qiu, Chunlai Nie, Yuquan Wei

**Affiliations:** State Key Laboratory of Biotherapy and Cancer Center, West China Hospital, Sichuan University, Chengdu, China; Institut Jacques Monod, France

## Abstract

**Background:**

Chemo-resistance to cisplatin-centered cancer therapy is a major obstacle to the effective treatment of human ovarian cancer. Previous reports indicated that arsenic trioxide (ATO) induces cell apoptosis in both drug-sensitive and -resistant ovarian cancer cells.

**Principal Findings:**

In this study, we determined the molecular mechanism of ATO-induced apoptosis in ovarian cancer cells. Our data demonstrated that ATO induced cell apoptosis by decreasing levels of phosphorylated AKT (p-AKT) and activating caspase-3 and caspase-9. Importantly, BIM played a critical role in ATO-induced apoptosis. The inhibition of BIM expression prevented AKT dephosphorylation and inhibited caspase-3 activation during cell apoptosis. However, surprisingly, gene silencing of AKT or FOXO3A had little effect on BIM expression and phosphorylation. Moreover, the activation of caspase-3 by ATO treatment improved AKT dephosphorylation, not only by cleaving the regulatory A subunit of protein phosphatase 2A (PP2A), but also by increasing its activation. Furthermore, our data indicated that the c-Jun N-terminal kinases (JNK) pathway is involved in the regulation of BIM expression.

**Conclusions:**

We demonstrated the roles of BIM in ATO-induced apoptosis and the molecular mechanisms of BIM expression regulated by ATO during ovarian cancer cell apoptosis. Our findings suggest that BIM plays an important role in regulating p-AKT by activating caspase-3 and that BIM mediates the level of AKT phosphorylation to determine the threshold for overcoming cisplatin resistance in ovarian cancer cells.

## Introduction

Ovarian cancer is the most common cause of cancer deaths from gynecologic tumors [1]. Cisplatin and its analogues are the key compounds of chemotherapy for human ovarian cancers, but chemo-resistance is a major obstacle hindering the successful treatment of ovarian cancer patients [2], [3]. Thus, it would be a major breakthrough in continued preclinical studies to find a new, low-toxicity, but efficient drug to overcome cisplatin resistance.

ATO, which has been proven to be an effective chemotherapeutic drug for the treatment of relapsed/refractory acute promyelocytic leukemia (APL) in the 1990s [4], has been approved by the FDA (Federal Drug Administration) for treating all-trans retinoic acid (ATRA)-resistant APL [5]. The remarkable efficacy of ATO in the treatment of APL has led to the exploration of its anticancer activity and underlying mechanism in other malignancies. There have been promising studies indicating that ATO not only possesses inherent single-agent tumoricidal activity against ovarian-cancer cell lines, but also can trigger apoptosis in cisplatin-resistant cells [6]–[10]. However, the precise molecular mechanisms by which ATO overcomes chemo-resistance and induces apoptosis in ovarian cancer cells are poorly understood.

Recent evidence suggests that the failure of drug-induced apoptosis may be an underlying cause of drug resistance. Some studies have identified a number of key mediators of apoptosis that are altered in chemo-resistant ovarian cancer cells [10]–[13]. The levels of expression and activation of the BCL-2 family proteins often play important roles in controlling apoptotic responses to drug treatments, thus modulating the chemo-sensitivity of tumor cells [14]–[16]. Overexpression of BCL-2 and BCL-XL genes contribute to apoptotic inhibition and the development of the multidrug-resistance of human ovarian cancers. The p53 protein is also a key regulator of chemo-sensitivity in ovarian cancer cells and is rapidly upregulated in response to DNA-damaging agents, such as cisplatin. Moreover, p53 induces apoptosis and regulates the release of cytochrome *c*, not only by modulating the transcription of the BH3-only protein (e.g., PUMA and NOXA), but also through a transcription-independent mechanism involving the direct translocation of the p53 protein to the mitochondria followed by inhibitory interactions with BCL-2 and BCL-XL [13], [17], [18].

BIM is also a member of the BH3-only family of pro-apoptotic proteins and is expressed in a wide variety of tissues [19], [20]. As determined by western blot analysis, most tissues express one predominant isoform of BIM, termed BIM-EL [21]. Following an apoptotic-stress event, BIM translocates to the mitochondria and initiates the mitochondrial cell death pathway by either directly activating BAX-like proteins or indirectly binding pro-survival BCL-2 family members, thereby releasing the BAX-like proteins [22], [23]. The BAX protein has been demonstrated to modulate apoptosis in ovarian cancer cells [2], [13]. However, the effect of BIM on cisplatin-sensitive and -resistant ovarian cancer cells has not been thoroughly elucidated.

Recent reports have indicated that AKT is an important determinant of cisplatin sensitivity in ovarian cancer cells. AKT promotes cell survival, suppresses apoptosis, and regulates cisplatin sensitivity in ovarian cancer cells [10]–[13]. Moreover, AKT, also known as protein kinase B or Rac [11], is an inactive cytosolic protein. AKT is recruited to the plasma membrane and activated by phosphorylation at threonine 308 and serine 473 in response to growth factors or cytokines [2], [10], [11], [24]. The molecule responsible for the recruitment of AKT to the cellular membrane, as well as its activation, is the phosphatidylinositol 3-kinase (PI-3K) [2], [11]. Both PI-3K and AKT are frequently activated and/or overexpressed in ovarian cancers [2], [11], [13], [25]. Upon activation, AKT phosphorylates several molecules involved in the regulation of apoptosis, such as the pro-apoptotic proteins BAD, BIM and caspase-9 and the transcription factor FOXO3A. Next, p-AKT blocks the binding of BAD or BIM to BCL-XL, inhibits caspase-9 protease activity, blocks FOXO3A function and reduces Fas ligand transcription [19], [26]–[28].

Previous studies have also demonstrated a modulating role of PI-3K/AKT signaling on BIM expression. The PI-3K inhibitor LY294002 increases BIM expression in cells concomitant with an increase in cell death [19]. However, it is proposed that an important downstream effect of LY294002 is to reduce the amount of AKT in its active, phosphorylated form [2], [19]. The link between reduced AKT activity and increased BIM expression levels is FOXO3A, a member of the forkhead family of transcriptional regulators that can directly increase BIM expression levels and induce apoptosis in cancer cells when overexpressed [19], [29], [30]. Thus, the upregulation of BIM expression, as regulated by LY294002, is connected with a loss of AKT activity and phosphorylation and an increase in the active form of FOXO3A.

Although the AKT-BIM pathway is important in the chemo-sensitivity and apoptosis of cancer cells, whether AKT regulates BIM activity in ovarian cancer cells during ATO treatment remains unclear. In our report, we showed that the expression level of BIM was increased and that the phosphorylation of AKT was decreased during the apoptosis of cisplatin-sensitive and -resistant cells after ATO treatment. Moreover, the downregulation of AKT by siRNA could not inhibit BIM expression and phosphorylation induced by ATO, whereas the knockdown of BIM prevented AKT dephosphorylation in ovarian cancer cells. Furthermore, our results demonstrated that ATO-induced AKT dephosphorylation is dependent on caspase-3-mediated PP2A activation, which is regulated by BIM expression. These results suggest that the phosphorylation of AKT is negatively modulated by BIM and that BIM-mediated AKT phosphorylation is a major molecular event in mediating ATO-induced apoptosis in chemo-sensitive and -resistant ovarian cancer cells.

## Results

### ATO induces apoptosis in cisplatin-sensitive and -resistant ovarian cancer cells

**Figure 1:**
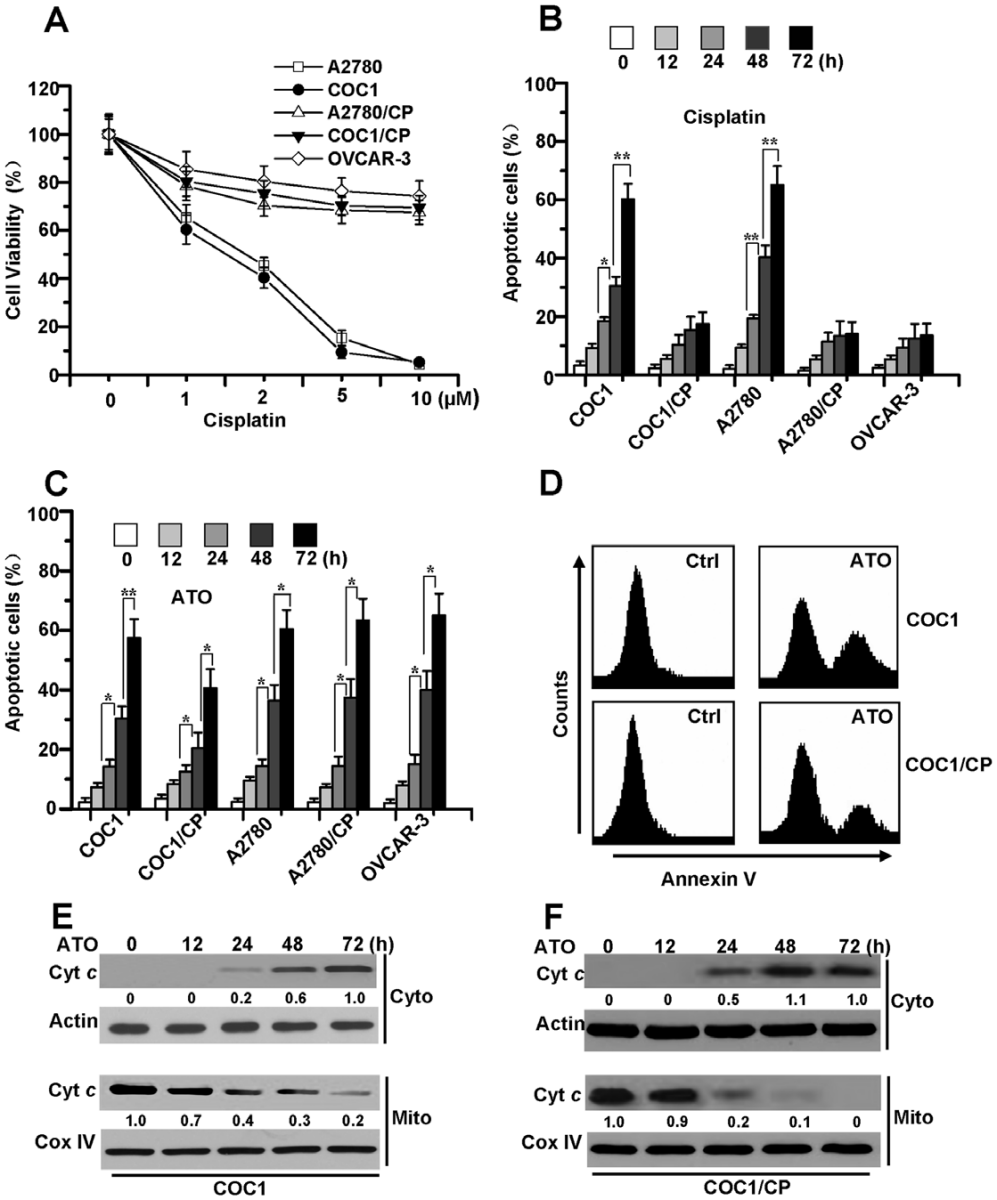
ATO induces apoptosis in cisplatin-sensitive and -resistant human ovarian cells. **A.** Dose-dependent effects of cisplatin in ovarian cell lines. Cells were exposed to cisplatin at the indicated concentrations in DMEM or RPMI-1640 with 10% FBS in 96 well plate for 72 h and cell viability was assessed by MTT assay. Graphs showing results of MTT assay. All data are depicted graphically as the means ± standard errors of the means for at least three independent experiments. **B.** Analysis of cell apoptosis. Cells were treated with cisplatin (5 µM) for different periods of time. Cell apoptosis was quantitatively detected by a cell death ELISA kit. Graphs showing results of quantitative analyses. All data are depicted graphically as the means ± standard errors of the means for at least three independent experiments. *, *P*<0.05, **, *P*<0.01. **C.** Effect of ATO on apoptotic death in cisplatin-sensitive and -resistant cells. Cells were cultured in the presence or absence of ATO (2 µM) for different periods of time, then cell apoptosis was measured as described in Materials and Methods. All data are depicted graphically as the means ± standard errors of the means for at least three independent experiments. *, *P*<0.05, **, *P*<0.01. **D.** Detection of cell apoptosis with flow cytometry analysis. COC1 and COC1/CP cells were treated with ATO (2 µM) for 48 h, then stained with Annexin V and examined by flow cytometry. **E** and **F.** Analyses of cyt *c* release. After treatment with ATO for different periods of time, cells were subjected to subcellular fractionation. The cytosolic or mitochondrial fractions were immunoblotted (30 µg of protein/lane) with antibody specific for cyt *c*. β-Actin and Cox IV were used as a protein loading control. Densitometric analysis of the Western blots was performed and the amount of cyt *c* was compared to the protein loading control. For cyto cyt *c*, relative amount of cyt *c* from treated cells (72 h) was set as 1, while relative amount from untreated cells (0 h) was set at 1 for mito cyt *c*. Data are representative of at least three independent experiments.

We first determined the growth-inhibitory effect of cisplatin in various cisplatin-sensitive (COC1 and A2780) and -resistant (COC1/CP, A2780/CP and OVCAR-3) human ovarian cell lines, as described previously [31]. Cell viability was detected by the MTT assay after 72 hours of treatment. As depicted in Figure 1A, cisplatin caused a dose-dependent reduction of cell viability in COC1 and A2780 cells, while COC1/CP, A2780/CP and OVCAR-3 cells demonstrably exhibited resistance to cisplatin. To examine cisplatin-induced apoptosis, the cells were treated with 5 µM cisplatin. Next, apoptosis was confirmed by a DNA fragmentation ELISA assay at various time points. These results demonstrated that cisplatin effectively induced apoptosis in cisplatin-sensitive ovarian cells, but not in cisplatin-resistant cells (Figure 1B). To investigate the effects of ATO on apoptosis in all ovarian cancer cells, we treated ovarian cancer cells with ATO and analyzed the cell apoptosis process by a DNA fragmentation ELISA assay. Our data suggested that ATO induced cell apoptosis in all ovarian cancer cells, regardless of their differences in chemo-sensitivity (Figure 1C). Flow-cytometry analysis with Annexin V staining further revealed that there was no difference in ATO-induced apoptosis between cisplatin-sensitive and -resistant cells (Figure 1D).

To determine whether mitochondria play an important role in ATO-induced cell apoptosis, the release of cytochrome *c* was detected by cell fractionation analysis. The results revealed that ATO induced the release of cytochrome *c* in a time-dependent manner in chemo-sensitive and -resistant cells (Figure 1E, F), suggesting that ATO initiates apoptotic cell death through mitochondrial dysfunction.

### BIM is important for ATO-induced apoptosis in chemo-sensitive and -resistant ovarian cancer cells

**Figure 2:**
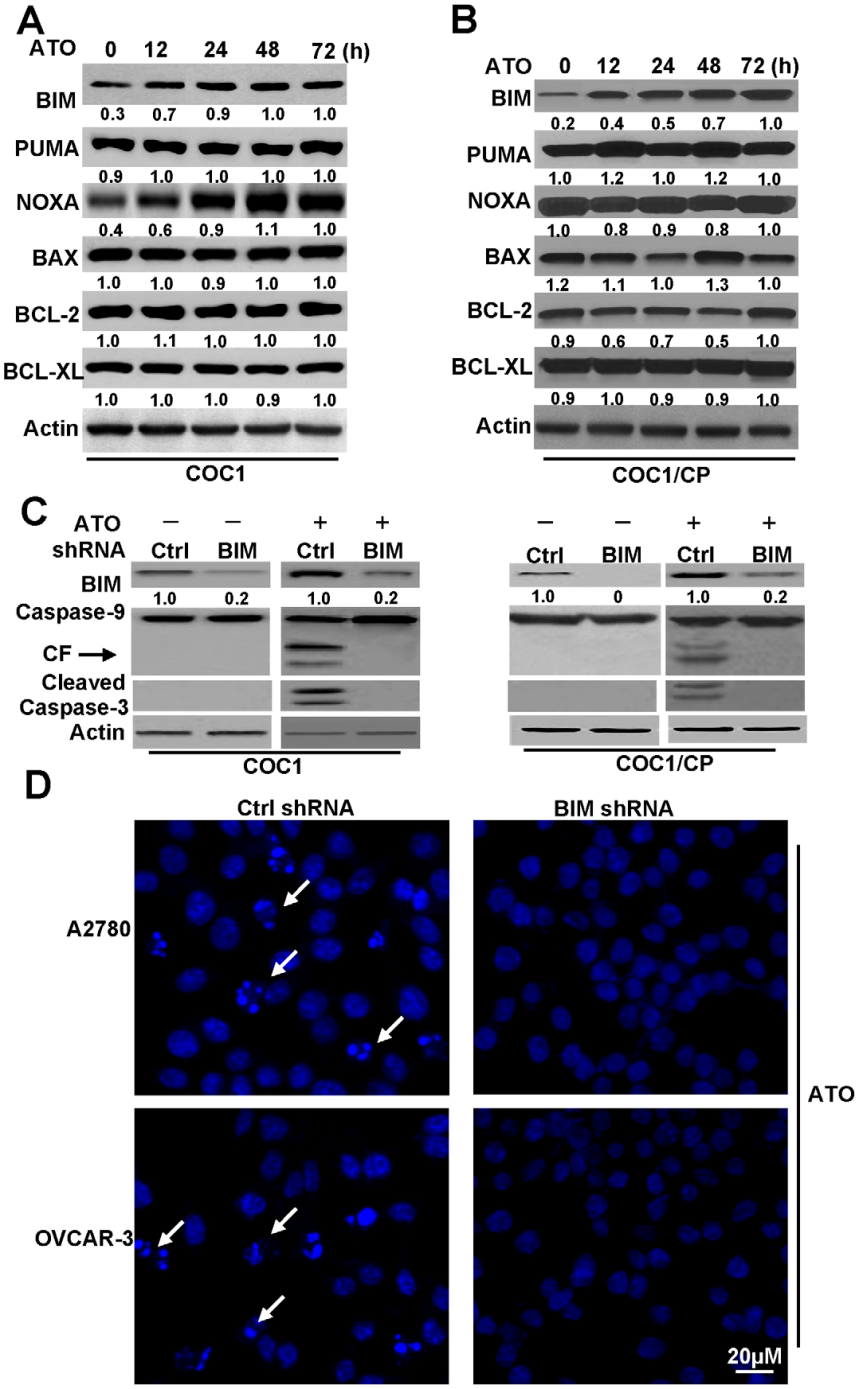
ATO regulated BIM expression is important for cell apoptosis in ovarian cancer cells. **A** and **B.** Western blots analysis of the expression level of BCL-2 family protein. Time-dependent analysis of BCL-2 member's expression levels in COC1/CP and COC1 cells. Cells were treated with ATO (2 µM) at indicated time, then lysed in NP40 buffer for detection. β-Actin was used as a protein loading control. Individual protein levels were measured by densitometric analysis of the Western blots and compared to actin levels. Relative amount of individual protein from treated cells (72 h) was set as 1. **C.** Effect of BIM shRNA on cell apoptosis. In the same conditions with **A**, cells were transfected with BIM or Ctrl shRNA for 48 h, and then treated with or without ATO for 48 h. Cell lysates were prepared and assayed for BIM, caspase-9 and cleaved caspase-3 by western blot. CF is referred to cleaved caspase-9. Relative fold means amount of BIM compared to actin and Ctrl condition was regarded as 1. **D.** Detection the effect of BIM shRNA on cell death with nuclear staining. Cells were transfected with BIM or Ctrl shRNA for 48 h, and then transfected cells were treated with ATO for 48 h. Cell death was assessed by measuring cells with condensed and fragmented nuclear by microscopy. Arrows indicate the condensed, fragmented, brightly stained nuclei, which are the hallmark of apoptosis. All data are representative of three independent experiments.

Mitochondrial dysfunction plays an important role in apoptosis in ovarian cells. Previous studies reported that changes in the gene expression of BCL-2-family proteins was involved in ATO-induced apoptosis [32] and that the BH3-only proteins were necessary for ATO-induced apoptosis in myeloma cells. However, it is unclear whether BH3 proteins might function in ovarian cancer cells following ATO treatment. Therefore, we investigated the expression of BCL-2-family proteins in cisplatin-sensitive and -resistant cells after ATO treatment. As depicted in Figure 2A, pro-apoptotic proteins, such as BAX, PUMA and NOXA, were not significantly changed in COC1 cells at various time points after ATO treatment. Anti-apoptotic BCL-2 and BCL-XL proteins also exhibited no major alterations. However, in contrast to other proteins, the expression level of BIM was markedly increased after ATO treatment, providing evidence that BIM was involved in apoptotic cell death in ovarian cancer cells. Meanwhile, ATO induced BIM expression equally in COC1/CP cells (Figure 2B). Similar results were also observed in other cell lines (data not shown). These results suggest that BIM plays an important role in ATO-induced apoptosis in ovarian cancer cells.

To confirm the effect of BIM on apoptosis in ovarian cancer cells, we performed gene-silencing experiments using an shRNA specific for BIM that targeted all known isoforms of its transcript. Similar to the result depicted in Figure 2A, the expression level of BIM was high in the control vector-transfected COC1 and COC1/CP cells after ATO treatment, while BIM expression levels in cells transfected with a BIM shRNA were relatively low (Figure 2C). Moreover, the downregulation of BIM inhibited caspase-9 and caspase-3 cleavage induced by ATO. Similarly, ATO induced nuclear fragmentation and condensation in control vector-transfected A2780 and OVCAR-3 cells, but not in BIM shRNA-transfected cells (Figure 2D). These results provide further evidence that BIM expression is necessary for ATO-induced apoptosis in ovarian cancer cells.

### AKT signaling is involved in cell death

**Figure 3:**
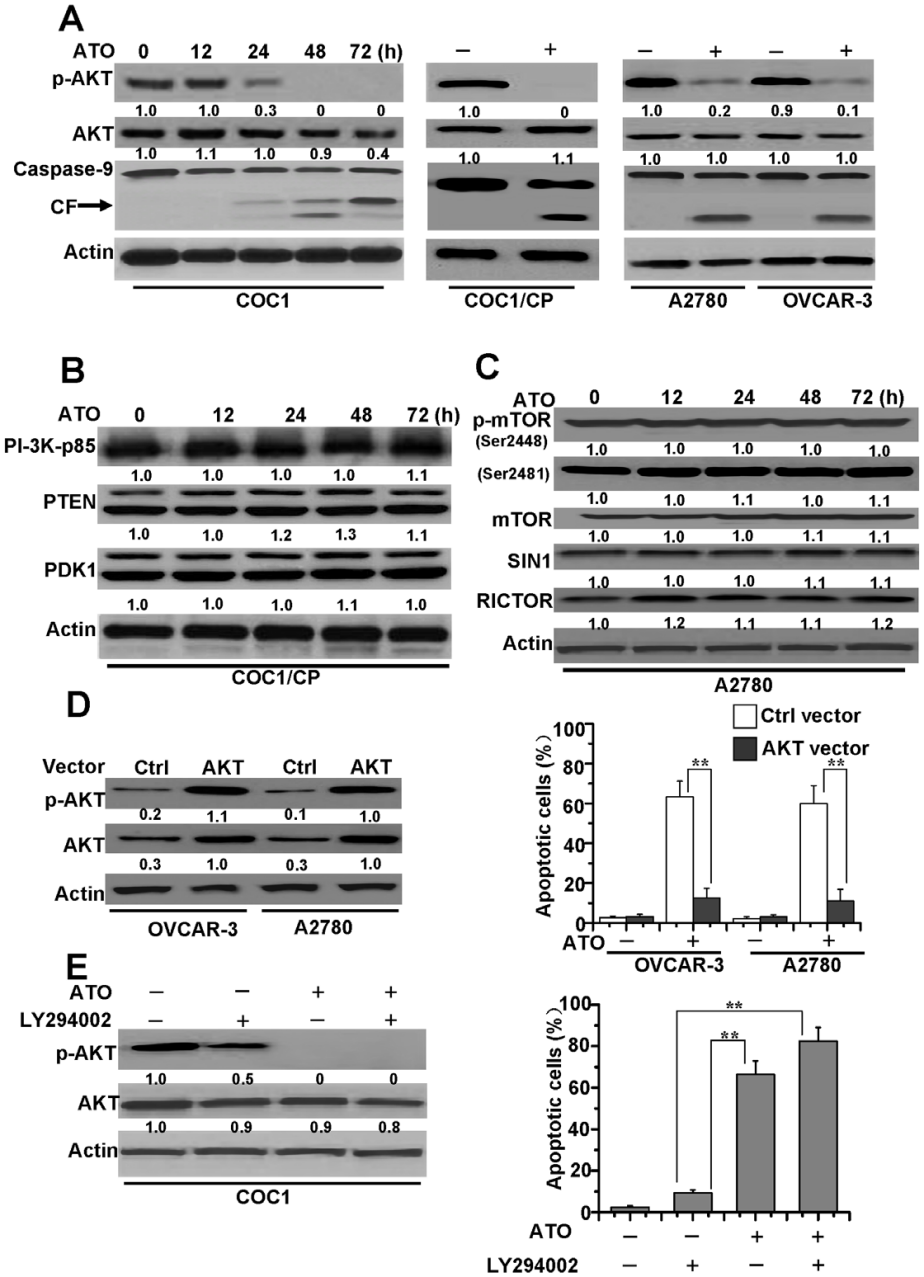
AKT is necessary for ATO-treated cell apoptosis. **A.** Detection of AKT phosphorylation, expression and caspase-9 activation. Western blot analysis of total AKT, p-AKT (Ser^473^) levels and caspase-9 cleavage in COC1 cells. Cells were treated with ATO (2 µM) at different time and lysed in NP40 buffer for detection. CF is referred to cleaved caspase-9. The same detection was employed in COC1/CP, A2780 and OVCAR-3. These cells were treated for 72 h, then lysed and assayed for individual protein levels by Western blot. β-Actin was used as a protein loading control. p-AKT and AKT levels were measured by densitometric analysis of the Western blots and compared to actin levels. Relative amount of p-AKT or AKT from untreated cells was considered as 1. **B.** Protein levels of the PI-3K signaling pathway in ATO-treated cells. Cells were exposed to drug for different periods of time. Cell lysates were prepared and assayed for individual protein levels by Western blot. Individual protein levels were measured by densitometric analysis of the Western blots and compared to actin levels. Relative amount of individual protein from untreated cells (0 h) was set as 1. **C.** Effects of ATO on protein levels of mTOR2, including p-mTOR (Ser ^2448^and ^2481^), mTOR, RICTOR, SIN1, in A2780 cells. Cells were exposed to drug as described in **B**, and then cell lysates were assayed by Western blot. Relative amount of protein levels were set as described in **B**. Cell lysates were assayed by western blot. Relative amount of protein levels were set as described in **B**. **D.** Protective effect of constitutive AKT on ATO-induced apoptosis in ovarian cells. OVCAR-3 and A2780 cells were transfected with constitutive AKT1 and were selected for 8 weeks by G-418 and treated with ATO for 72 h. Cells were lysed and assayed for individual protein levels by Western blot. *Left,* protein levels of p-AKT (Ser^473^) and AKT (AKT1) in both Ctrl or AKT vector transfected OVCAR-3 and A2780 cells were detected. Relative amount of protein levels were set as described in **B**. p-AKT and AKT levels of AKT1 transfected A2780 cells were considered as 1. *Right,* analysis of apoptosis in Ctrl or AKT1 vector transfected OVCAR-3 and A2780 cells. Cell apoptosis was quantitatively detected by a cell death ELISA kit as described in Materials and Methods. All data are depicted graphically as the means ± standard errors of the means for at least three independent experiments. **, *P*<0.01. **E.** Detection of the role of LY294002 on apoptosis of cells. Cells were treated with ATO and/or 25 µM LY294002 for 72 h, and then cells were lysed and assayed for individual protein levels by Western blot. *Left,* protein levels of p-AKT (Ser^473^) and total AKT in COC1 cells were detected. Cell apoptosis was quantitatively detected by a cell death ELISA kit. Relative amount of protein levels were set as described in **B**. p-AKT and AKT levels from untreated cells were set as 1. *Right,* analysis of apoptotic cells as describe in Materials and Methods. All data are depicted graphically as the means ± standard errors of the means for at least three independent experiments. **, *P*<0.01.

Recent studies have indicated that AKT modulates BIM activation either directly or indirectly [19], [33]. Moreover, ATO induces apoptosis in myeloma cells by decreasing AKT activity and expression in cells [34]. Based on the above observations, we speculate that AKT is probably involved in ATO-induced apoptosis by regulating BIM expression. Accordingly, we first measured whether AKT is involved in ATO-induced apoptosis in ovarian cancer cells. As shown in Figure 3A, ATO induced the downregulation of p-AKT at Ser473 in cisplatin-sensitive COC1 cells in a time-dependent manner, although the expression level of total AKT protein exhibited no change. Downregulation of p-AKT induced activation of caspase-9 in sensitive COC1 cells. Similarly, ATO induced the reduction of p-AKT and the cleavage of caspase-9 in other ovarian cancer cells, in spite of their differences in chemo-sensitivity.

We then analyzed the expression levels of upstream PI-3K signaling proteins, which might affect p-AKT levels in ovarian cancer cells. As depicted in Figure 3B, levels of PI-3K pathway proteins, including p85 (the regulatory subunit of PI-3K), PTEN and PDK1 were not altered significantly after ATO treatment for various periods of time in COC1/CP cells. Recent studies suggested that the mammalian target of rapamycin 2 (mTORC2) or the Rictor protein was responsible for the activation of p-AKT at Ser473 [35], [36], and the depletion of subunits of intact mTORC2, such as SIN1, abolished AKT phosphorylation at Ser473. Moreover, Ser2448 on mTOR was an AKT phosphorylation site, and phospho-Ser2481 was a marker of mTORC2 complexes [35], [37]. Our data demonstrated that the protein levels of mTOR complex 2, including phospho-mTOR (at Ser 2481 and 2448), mTOR, RICTOR and SIN1 were not altered significantly in A2780 cells after exposure to ATO at various periods of time (Figure 3C). These results suggest that these proteins have no effect on the p-AKT level.

To validate if AKT signaling is a major molecular target responsible for ATO-induced apoptosis, we transfected a constitutively active AKT1 gene into OVCAR-3 and A2780 cells and examined their response to ATO treatment. As presented in Figure 3D, OVCAR-3 and A2780 cells transfected with AKT1 exhibited obvious resistance to ATO-induced apoptosis.

Moreover, we used LY294002, a PI-3K inhibitor, to inhibit the phosphorylation of AKT by upstream signaling molecules and examined the pro-apoptotic effect of ATO in ovarian cancer cells. We found that ATO alone eliminated p-AKT and induced apoptosis while LY294002 alone was not sufficient to induce apoptosis, though it partially decreased p-AKT. Further experiments proved that the combination of LY294002 and ATO completely eliminated p-AKT and enhanced induction of apoptosis in COC1 cells (Figure 3E). Our data indicates that the effect of combination of LY294002 and ATO on p-AKT and apoptosis is more significant than that of LY294002 alone, and that the AKT signaling pathway is involved in ATO-induced apoptosis in ovarian cancer cells.

### BIM-regulated AKT phosphorylation in cell apoptosis

**Figure 4:**
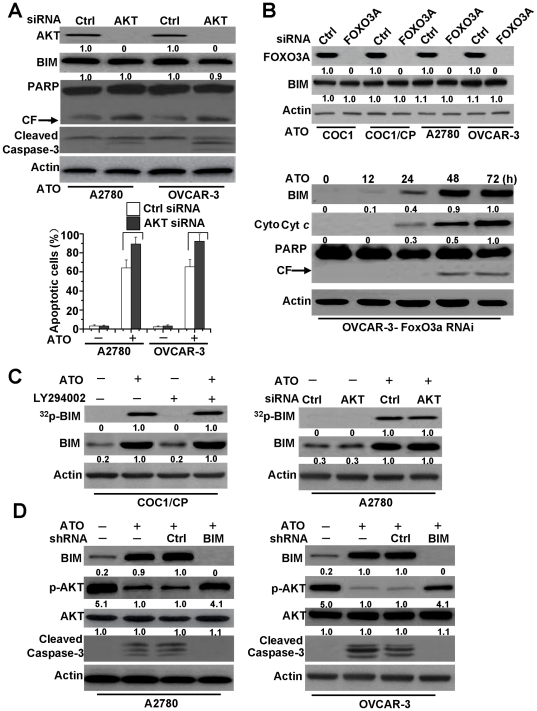
BIM mediated-AKT dephosphorylation regulates apoptosis induced by ATO. **A.** Effect of AKT siRNA on the BIM expression. Cells were transfected with either control siRNA or AKT1 siRNA for 48 h and then were exposed to ATO for 72 h. Cells were lysed and assayed for individual protein levels by Western blot. CF is referred to cleaved PARP. β-Actin was used as a protein loading control. *Up,* protein levels of AKT, BIM, cleaved caspase-3, and PARP were detected by Western blot. Relative amount of individual protein level was set as described in **Figure 3B**. p-AKT and AKT levels of Ctrl siRNA transfected and treated A2780 cells were considered as 1. *Down,* analysis of apoptotic cells. as described in Material and Methods. All data are depicted graphically as the means ± standard errors of the means for at least three independent experiments. *P*>0.05 (not significant). **B.** Effect of FOXO3A siRNA on the BIM expression and cell apoptosis. Cells were transfected with either control siRNA or FOXO3A siRNA for 48 h and then were exposed to ATO for 72 h. *Up,* Following transfection for 48 h, Immunoblot for BIM and FOXO3A protein expression was performed on whole cell lysates. Relative amount of individual protein level was set as described in **Figure 3B**. FOXO3A and BIM levels of Ctrl siRNA transfected COC1 cells were considered as 1. *Down,* protein levels of BIM, cytosol cyt *c* and PARP were assayed by Western blot in FOXO3A siRNA OVCAR-3 cells. For cyt *c* detection, cells were treated following **Figure 1E**. As described in **Figure 3B**, relative amount of BIM and cyt *c* in ATO treated cells (72 h) were regarded as 1. **C.** Effect of AKT on BIM phosphorylation. Cells were metabolically labeled with [^32^P] orthophosphoric acid and treated with ATO (2 µM) for 48 h, and BIM was immunoprecipitated by using an agarose-conjugated BIM antibody, then detection of phosphorylation of BIM. Phosphorylation of BIM was determined by autoradiography (upper panel). Western blot analysis was performed to confirm and quantify BIM protein (lower panel). *Left,* the effect of LY294002 on BIM phosphorylation. Cells were treated with ATO and/or LY294002 (25 µM), then detection of BIM expression and phosphorylation. As described in **Figure 3B**, relative amount of ^32^p-BIM and BIM in ATO treated cells were regarded as 1. *Right,* the effect of AKT siRNA on BIM phosphorylation. Cells were transfected with either control siRNA or AKT1 siRNA for 48 h and then were exposed to ATO for 48 h. Detection of BIM expression and phosphorylation as described. Relative amount of ^32^p-BIM and BIM in AKT transfected and ATO treated cells were set as 1. **D.** Effect of BIM shRNA on AKT phosphorylation. Cells were transfected with either control shRNA or BIM shRNA for 48 h and then were exposed to ATO for 48 h. Western blot examined BIM expression, caspase-3 cleavage, protein levels of p-AKT (Ser^473^) and AKT in cells. Relative amount of p-AKT, AKT and BIM in Ctrl shRNA transfected and ATO treated cells were set as 1. All data are representative of three independent experiments.

AKT mediates BIM activation through two primary pathways: (1) AKT phosphorylates BIM directly and inhibits BIM activation [19], [33], or (2) AKT phosphorylates FOXO3A, leading to its cytoplasmic retention by 14-3-3 proteins. Thereby, AKT could not translocate into the nucleus to induce BIM transcription, meaning that AKT regulates BIM activation indirectly. On the basis of these observations, we decided to examine whether AKT affects BIM activation in ovarian cancer cells. We first downregulated the expression of AKT1 by siRNA in ATO-treated A2780 and OVCAR-3 cells and found that the downregulation of AKT increased caspase-3 and PARP cleavage during ATO treatment. However, AKT downregulation had little effect on BIM expression, though AKT downregulation increased, to some extent, ATO-induced nuclear fragmentation and followed cell apoptosis (Figure 4A). Similar results were also found in ATO-treated ovarian cancer cells when FOXO3A expression was downregulated using siRNAs targeting FOXO3A (Figure 4B). These results suggest that the AKT pathway may not be involved in regulating BIM expression during ATO-induced apoptosis.

To detect BIM phosphorylation during apoptosis, A2780 and COC1/CP cells were metabolically labeled with [^32^P] orthophosphoric acid and treated with ATO for 48 h, and the phosphorylation of BIM was analyzed by autoradiography. A previous report demonstrated that BIM induction and phosphorylation increased during apoptosis [38]. Our studies also have examined BIM phosphorylation and increased BIM expression after ATO treatment. Our data further revealed that AKT downregulation by siRNA or an AKT inhibitor (LY294002) could not decrease BIM phosphorylation in chemo-sensitive and -resistant cells after ATO treatment (Figure 4C). These results indicated that AKT inactivation had little to no effect on BIM phosphorylation and suggested that AKT could not regulate BIM activation and that other potential mechanisms regarding the regulation of BIM activation may exist.

However, our further data indicated that knockdown of BIM inhibited AKT dephosphorylation and caspase-3 cleavage in chemo-sensitive and-resistant ovarian cancer cells (Figure 4D). These results suggest that ATO-induced BIM expression prevents the phosphorylation of AKT, indicating that BIM regulates AKT activation during ATO stimulation in ovarian cancer cells.

### BIM regulates AKT dephosphorylation by caspase-3 activity

Previous reports indicated that AKT was dephosphorylated at both Thr308 and Ser473 and inactivated in vitro by the protein phosphatase 2A (PP2A), which formed a complex with AKT. AKT was activated in cells upon treatment with the PP2A inhibitors, such as okadaic acid (OA) [39], [40]. Recently, it was reported that caspase-3 regulated the phosphorylation of AKT through the cleavage of the regulatory A subunit of PP2A (PP2A/A), which increased PP2A activity [39]. Our results demonstrated that BIM is involved in the regulation of caspase-3 and AKT activation (Figure 4D). Therefore, we propose that BIM may regulate AKT phosphorylation by caspase-3-mediated PP2A activation in ovarian cells.

**Figure 5:**
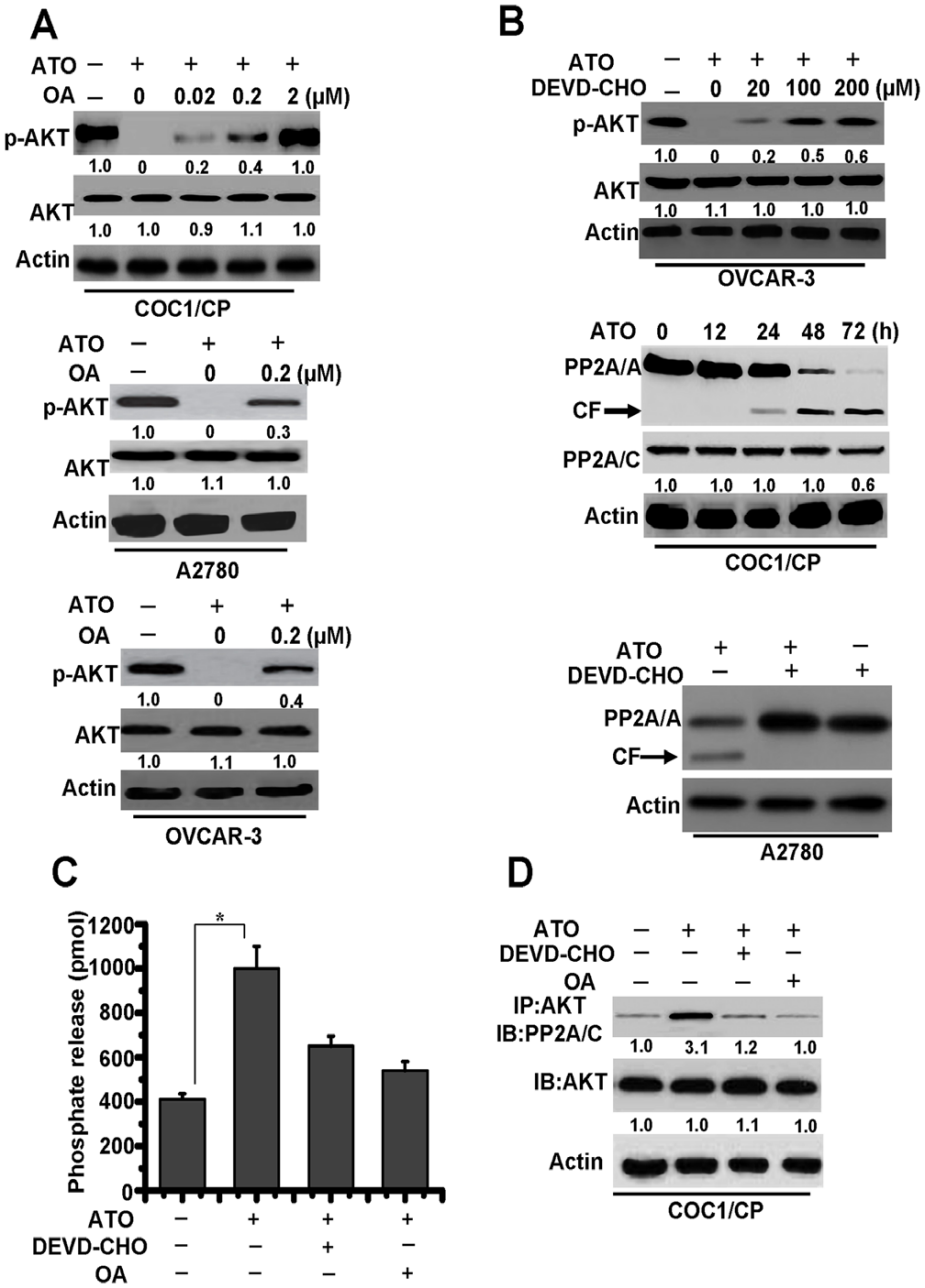
Caspase-3 mediates PP2A-related AKT dephosphorylation. **A.** Different cells were pre-incubated with indicated concentrations of OA for 1 h and exposed to ATO for 48 h, then lysed in NP40 buffer for detection. Western blot analysis of total AKT and p-AKT (Ser^473^) levels was shown. β-Actin was used as a protein loading control. Relative amount of p-AKT and AKT in untreated cells were set as 1. **B.** Analysis of the effect of caspase-3 on PP2A activation. *Up*, Cells were pre-incubated with indicated concentrations of DEVD-CHO for 1 h and exposed to ATO for 48 h, then lysed in NP40 buffer for detection. Western blot analysis of total AKT and p-AKT (Ser^473^) levels was shown. Relative amount of p-AKT and AKT in untreated cells were set as 1. *Middle*, the treated cells were lysed with sample buffer and subjected to immunoblot assay with an A subunit-specific anti-PP2A/A antibody. CF is referred to cleaved PP2A/A. The membrane was then stripped and reprobed with a C subunit-specific anti-PP2A/C antibody. Relative amount of PP2A/C in untreated cells (0 h) were set as 1. *Down*, Western blot analysis of the effect of DEVD-CHO (100 µM) in PP2A cleavage. **C.** Detection of PP2A activity. Cells were pre-incubated with or without 0.2 µM of OA or 100 µM of DEVD-CHO for 1 h and exposed to drug for 48 h. PP2A activity with the cellular proteins extracted from RIPA buffer-lysed cells was measured using phosphopeptide KIpTIRR as a substrate as described in Materials and Methods. Each column represents the mean ± S.D. of triplicate assays. *, *P*<0.05. **D.** Detection of the binding of AKT with PP2A. Cells were incubated with or without ATO for 48 h. In some groups of cells, 0.2 µM of OA or 100 µM of DEVD-CHO was added 1 h prior to the addition of drug. The cells were then lysed with RIPA buffer for immunoprecipitation with anti-AKT antibody followed by immunoblot assay with anti-PP2A/C and anti-AKT antibodies. Relative amount of individual protein in untreated cells were set as 1. Data are representative of at least three independent experiments.

To validate our hypothesis, we first determined whether PP2A mediated AKT phosphorylation in our study. Pretreatment of the cells with OA, an inhibitor of PP2A, resulted in a gradual reversal of ATO-induced AKT dephosphorylation in a dose-dependent manner in COC1/CP cells (Figure 5A). At the same time, OA also rescued AKT phosphorylation in A2780 and OVCAR-3 cells during ATO treatment.

To determine whether caspase-3 mediates AKT phosphorylation and the proteolysis of the A subunit linked to the increased PP2A activity, we pre-treated cells with DEVD-CHO, a specific caspase-3 inhibitor. The results indicated that inhibition of caspase-3 activation restored AKT phosphorylation in the presence of ATO at various periods of time and inhibited PP2A proteolysis. Obvious cleavage of the A subunit, not the catalytic C subunit; was observed at time points tested from 24 to 72 h without DEVD-CHO (Figure 5B), suggesting that ATO-mediated activation of PP2A may primarily be involved in the caspase-mediated degradation of the A subunit.

To confirm the involvement of caspase-3 in PP2A activation, PP2A activity was measured by detecting the release of phosphate from the synthetic PP2A substrate KIpTIRR [39]. ATO treatment caused an increase of phosphate release, indicating elevated PP2A activity, which was blocked by pre-treating the cells with the indicated concentration of OA or DEVD-CHO (Figure 5C). In addition, as shown in Figure 5D, a low but detectable quantity of the catalytic C subunit of PP2A (PP2A/C) was co-precipitated with AKT in untreated cells, indicating the occurrence of a physiological association between these two proteins. Interestingly, the PP2A-AKT association was greatly improved after a 48 h treatment of ATO, whereas this interaction was drastically decreased by the inhibition of caspase-3 and PP2A activation, suggesting that the dephosphorylation of AKT is a consequence of caspase-mediated PP2A activation and that the caspase-3-PP2A-AKT pathway is a positive feedback loop on apoptosis. These results also indicate that BIM-caspase-AKT may represent a new pathway which regulates ATO-induced apoptosis in ovarian cancer cells.

### The JNK pathway mediates BIM activation in cell apoptosis

**Figure 6:**
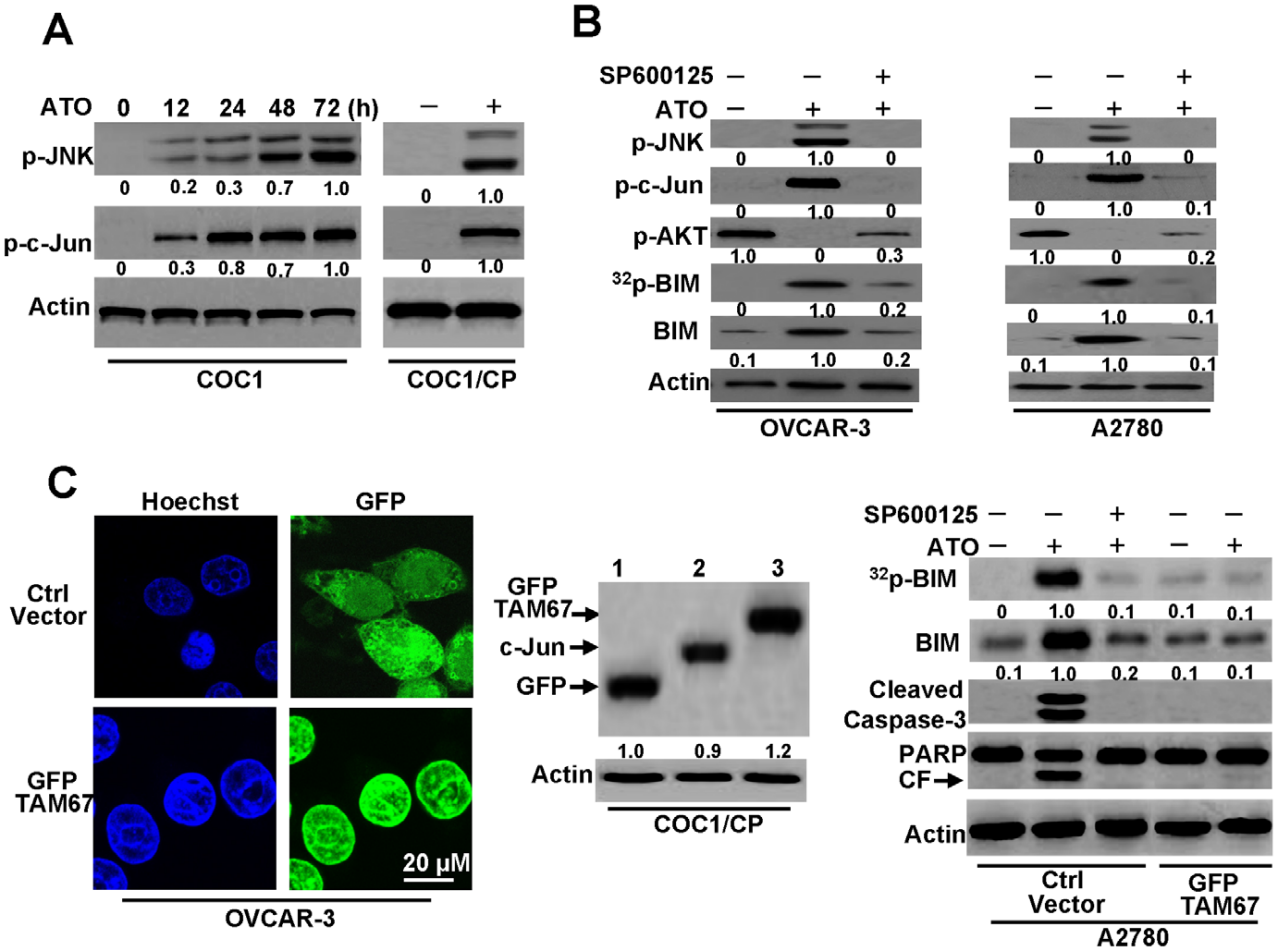
JNK pathway upregulates BIM expression and phosphorylation. **A.** Detection of p-JNK, and p-c-Jun in cells. COC1 cells were treated with ATO (2 µM) for different periods of time, then lysed with sample buffer and subjected to immunoblot assay with antibodies for phosphor-c-Jun (Ser 63) or polyclonal-phospho-JNK. COC1/CP cells were treatd with ATO for 72 h, then lysed for detection. Relative amount of p-JNK and p-c-Jun in untreated cells (72 h) were set as 1. **B.** JNK inhibitor prevents BIM expression and phosphorylation. Cells were treated with ATO (2 µM) for 72 h in the presence of JNK inhibitors SP600125 (10 µM). Lysated cells were immunoblotted with antibodies for phospho-c-Jun (Ser 63), polyclonal-phospho-JNK, phospho-AKT (Ser 473) or polyclonal-BIM. BIM phosphorylation was detected as described in **Figure 4**. Relative amount of p-JNK, p-c-Jun, ^32^p-BIM and BIM in ATO treated cells were set as 1. Relative fold means amount of p-AKT from untreated cells were regarded as 1. **C.** Dominant negative c-Jun inhibits BIM expression and phosphorylation. *Left,* OVCAR-3 cells were transiently transfected with pEGFP-TAM67 mutant or control vector (Ctrl vector). The mutant location was detected by microscopy. *Middle,* COC1/CP cells were transfected with pEGFP-C2 or pEGFP-TAM67, then lysed with antibodies for GFP or c-Jun. lane 1: cells with pEGFP-C2 vector. 2: untransfected cells. 3: cells with pEGFP-TAM67. Relative fold means amount of GFP in pEGFP-C2 vector transfected cells was regarded as 1. *Right,* A2780 cells were transfected with pEGFP-TAM67 or Ctrl vector. Stable positive clones were selected with 1 mg/ml G418 for several weeks. Ctrl vector transfected cells were treated with ATO (2 µM) and/or SP600125 for 72 h. pEGFP-TAM67 transfected cells were also incubated with ATO for 72 h. Lysated cells were immunoblotted with antibodies. BIM phosphorylation was detected as described in **Figure 4**. In all immunoblot analysis, β-Actin was used as a protein loading control. Relative amount of ^32^p-BIM and BIM in ATO treated cells were set as 1. All data are representative of three independent experiments.

Although we have confirmed that BIM mediates AKT activation in ATO-induced apoptosis, the mechanism of how ATO induces BIM is obscure. Recent studies demonstrated that ATO triggered the JNK pathway in cell apoptosis [41] and that JNK activation modulated BIM phosphorylation and expression [42], [43]. Accordingly, we wondered if JNK is activated and mediates BIM activation during ATO-induced apoptosis in ovarian cancer cells. To determine the function of the JNK pathway in BIM activation during ATO-induced apoptosis, we analyzed the phosphorylation of JNK (p-JNK) and c-Jun (p-c-Jun). The results demonstrated that ATO triggered the phosphorylation of JNK and c-Jun in a time-dependent manner, suggesting that the JNK pathway is activated during ATO-induced apoptosis (Figure 6A). In addition, our data also revealed that JNK activation increased BIM expression and phosphorylation, while the JNK inhibitor SP600125 prevented BIM activation (Figure 6B). These results suggest that the JNK pathway contributes not only to BIM expression, but also to the phosphorylation of BIM.

To further confirm that the JNK signaling pathway mediates BIM activation and subsequent cell apoptosis, we utilized the dominant negative mutant of c-Jun, TAM67, which was cloned into the pGFP-C2 plasmid. We first examined the location and expression of GFP-TAM67 in cells. Our data showed that GFP-TAM67 was located in the nucleus, which is consistent with results described previously [44]–[46]. An immunoblotting assay also demonstrated the expression of GFP-TAM67 in transfected cells (Figure 6C). Previous studies demonstrated that TAM67 inhibited the JNK pathway-mediated activation of BIM and subsequent caspase cleavage [44], [45]. Furthermore, our results revealed that dominant-negative c-Jun not only inhibited BIM phosphorylation and expression, but also repressed caspase activation. Therefore, these results confirm that JNK signaling promotes BIM activation during ATO-induced apoptosis.

## Discussion

ATO, which is used effectively to treat patients with APL, not only shows promise as a treatment for multiple cancers, but has multiple interactions that may help overcome the cisplatin resistance of some cancer cells [47]. Understanding the mechanism by which ATO induces apoptosis may identify potential targets for combination therapies of malignancies. In this study, we demonstrated that ATO can induce apoptosis in ovarian cancer cell lines, regardless of their differences in chemo-sensitivity (Figure 1) [6]. The importance of our findings is that we identified the precise molecular mechanisms of apoptosis in ovarian cancer cells induced by ATO. We determined that BIM is a key mediator during ATO-induced apoptosis by detecting the expression levels of BCL-2-family proteins in cells. It was reported that BIM downregulation is important for cell survival, as expression of this death activator at levels equivalent to those induced by cytokine withdrawal led to apoptosis, even in the presence of IL-3 [33], and the silencing of BIM also protected cells from ATO-induced apoptosis, indicating that upregulation of the BH3-only proteins contributes to ATO-induced toxicity [32]. Therefore, our studies further confirmed the important roles of BIM in ATO-induced apoptosis, as our results demonstrate that BIM downregulation by shRNA inhibits ATO-induced cell apoptosis.

Several labs have reported that AKT plays a role in ATO-induced cell death [34], [48]. Pre-treatment with PI-3K pathway inhibitors enhanced ATO-induced apoptosis in leukemia cells, and the cytotoxic effect of ATO correlated with a decrease in p-AKT, but not total AKT [48]. In addition, the PI-3K/AKT signaling pathway played a critical role in the carcinogenesis and drug resistance exhibited by numerous types of cancer cells, including ovarian cancer cells [12], [13], [49], supporting the importance of PI-3K/AKT signaling in the apoptosis of ovarian cancer cells. Previous reports also demonstrated that BIM activation is dependent on the dephosphorylation of AKT and indicated the ability of the PI-3K inhibitor LY294002 to induce BIM expression and apoptosis. Recent studies have suggested that the link between reduced AKT activity and increased BIM expression levels is FOXO3A, a member of the forkhead family of transcriptional regulators that can directly elevate BIM expression levels and induce apoptosis in cells. FOXO3A is phosphorylated by AKT, leading to its cytoplasmic retention by 14-3-3 proteins and loss of target-gene activation [24], [33], [50]. These findings suggest that AKT is likely to be involved in regulating BIM expression in ovarian cancer cells' response to ATO stimulation.

Although our data suggested that p-AKT is necessary for ATO-induced apoptosis, AKT downregulation by RNAi, surprisingly, could not prevent BIM expression during ATO treatment. Additionally, FOXO3A inhibition by RNAi had little effect on BIM expression. Moreover, ATO treatment induced an increase in BIM phosphorylation, even in cells transfected with AKT RNAi. These results implied that the AKT pathway is not involved in BIM activation. In contrast, our report suggests that BIM activation is involved in downregulating p-AKT during ATO-induced apoptosis. To our knowledge, the observation that BIM can regulate the phosphorylation of AKT is a novel finding.

**Figure 7:**
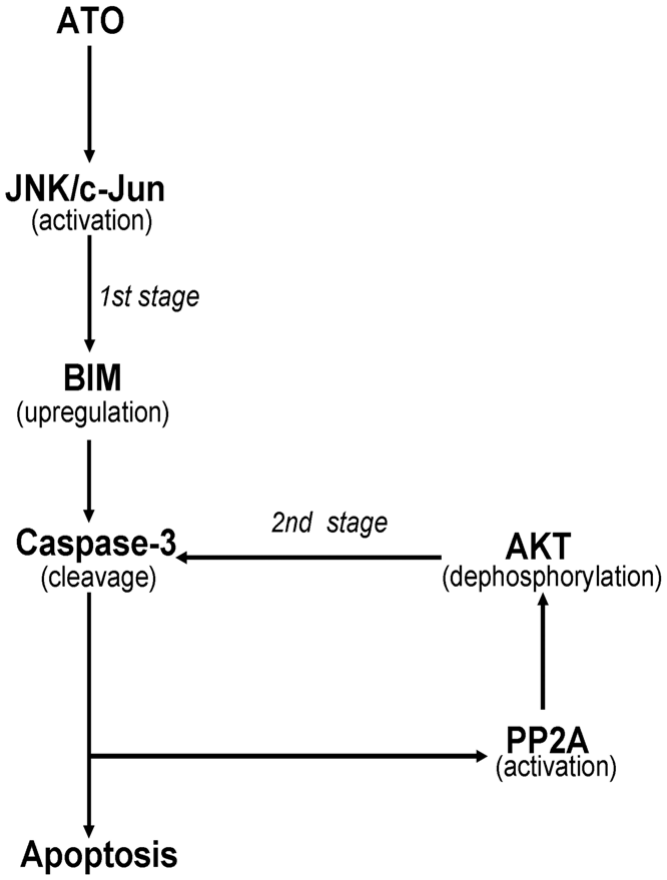
A diagram of signaling pathway for ATO-mediated BIM activation and apoptosis in ovarian caner cells. ATO induced JNK pathway activation, and then activated JNK regulated BIM activation. BIM expression upregulated caspase-3 activation and subsequent apoptosis at the first stage. Activated caspase-3 triggered PP2A-linked AKT dephosphorylation, and then AKT dephosphorylation further enhanced caspase-3 cleavage and apoptosis at the second stage.

In this study, we attempted to determine how BIM controls the phosphorylation of AKT during ATO-induced ovarian cancer cell apoptosis and which factors mediate BIM expression during cell apoptosis. We first examined the mechanism of p-AKT, as regulated by BIM. We observed that BIM shRNA could prevent caspase-3 cleavage, suggesting that BIM expression is an upstream event in caspase-3 activation. PP2A-linked, caspase-dependent pathways were required for downregulation of AKT kinase [34], [39], [40]. In this study, we also observed that AKT phosphorylation was regulated by caspase-3-mediated PP2A activation. Our results further confirm that BIM-mediated AKT phosphorylation requires two stages. In the first stage, BIM expression is regulated during ATO-induced apoptosis, and activated BIM triggers caspase-3 cleavage and subsequent events. In the second stage, caspase-3 activation mediates AKT dephosphorylation, further enhancing caspase-3 activation and ATO-induced apoptosis through a positive-feedback regulation (Figure 7). Subsequently, we detected which factors regulate BIM activation. A previous study suggested that ATO induces the JNK pathway activation in apoptosis [41]. In fact, activated JNK could modulate BIM expression and phosphorylation [42], [43]. Accordingly, we propose that JNK may participate in regulating BIM expression and phosphorylation during ATO-induced ovarian cancer cell apoptosis. Our data support the hypothesis that JNK activation triggers BIM expression and phosphorylation in ATO-induced apoptosis. Moreover, JNK activation was observed to be an early response to ATO treatment and was required for subsequent apoptotic events in cells. These result were consistent with a recent report from García-Pardo's group [51]. Their study indicated that ATO induces JNK activation, resulting in inhibition of the PI-3K/AKT signaling pathway in B-CLL cells and upregulation of PTEN. However, our results did not demonstrate PTEN upregulation during ATO-induced apoptosis. This discrepancy regarding the process of PTEN induction requires further research.

In this study, we explicate the molecular mechanism of ATO in ovarian cancer cells and provide the first evidence that BIM-mediated AKT dephosphorylation is an important factor for ATO-induced apoptosis in cisplatin-sensitive and -resistant ovarian cancer cells. The new JNK-BIM-AKT pathway may explain why ATO can induce cell apoptosis in cisplatin-resistant ovarian cancer cells (Figure 7). To understand the events involved in ATO-induced apoptosis will require us to design combinations of various agents with modest activities for uses as single agents in treating tumors.

## Materials and Methods

### Materials

Cisplatin, arsenic trioxide, Hoechst33342, LY294002, SP600125, DEVD-CHO and monoclonal anti-actin were obtained from Sigma (St. Louis, MO). Monoclonal anti-AKT1, polyclonal anti-AKT, polyclonal anti-phospho-AKT (Ser 473), anti-phospho-PI-3K (Ser 85), anti-phospho-c-Jun (Ser 63), polyclonal anti-phospho-JNK, polyclonal anti-mTOR, polyclonal anti-phospho-mTOR (Ser 2481 and 2448), polyclonal anti-BIM, anti-PP2A/A, anti-PP2A/C, monoclonal anti-caspase-9 and anti-cleaved caspase-3 were purchased from Cell Signaling (Beverly, MA). Polyclonal anti-PTEN, anti-RICTOR, anti-PDK1, anti-SIN1, anti-cytochrome *c*, anti-PARP, polyclonal anti-BAX, PUMA, NOXA, BCL-2 and BCL-XL were obtained from Santa Cruz (Santa Cruz, CA). The dominant-negative c-Jun plasmid (pEGFP-TAM67) was generously provided by Dr. Jian Zhao (Sichuan University, Chengdu, China).

### Gene Silencing with Small Interfering RNAs

The small interfering RNA (siRNA) duplexes targeting the sequence 5′-UAAUGUGCCCGUCCUUGUCUU-3′ of the human AKT1 gene, the sequence 5′-ACUCCGGGUCCAGCUCCAC-3′ of the FOXO3A gene and control siRNA oligonucleotides were purchased from Dharmacon Research, Inc. (Lafayette, CO). The BIM shRNA construct was a gift from Subhas C. Biswas (Columbia University College of Physicians and Surgeons, New York, USA) [52].

### Cell Culture and Transfection

Cells were obtained from the American Type Culture Collection (ATCC). COC1 and OVCAR-3 cells were cultured in suspension with RPMI-1640 media (Sigma, St. Louis, MO, USA) supplemented with 10% fetal bovine serum (FBS) (Hyclone) and 1% penicillin-streptomycin at 37 °C under 5% CO_2_. A2780 cells were incubated in DMEM media supplemented with 10% FBS and 1% penicillin-streptomycin. Cisplatin-resistant cells were obtained by reiterating treatments with increasing concentrations of cisplatin. These cell lines were grown as described previously [53]. Briefly, ovarian cells have been reproducibly obtained by exposing the cells to 5 µg/ml cisplatin under the same conditions as described above. Despite massive cell death concurrent with treatment, the cultures were maintained for four to six weeks by regular changes of culture medium until the drug-surviving cells recovered a normal growth pattern. Next, cisplatin treatment was reiterated with elevated concentrations (7.5-10 µg/ml). After establishment, the chemo-resistant variants were treated with cisplatin every month to maintain their high level of chemo-resistance.

For transfection, cells were placed on 6-well plates and transfected with the appropriate plasmid DNA or siRNA using the protocol described by the manufacturer. Typically, 1 µg of plasmid DNA or siRNA and 4 µl of DMRIE-C reagent (Invitrogen) were used per coverslip. The cells were incubated for 4 h in the transfection mixture, which was then replaced with fresh culture medium. Positive clones were selected with 1 mg/ml G418 for several weeks. For overexpression of AKT, cells were transfected with the constitutively active AKT1 construct HA-PKB-T308D/S473D as previously described [24].

### Cell Viability and Apoptosis Assay

Ovarian**-**cell viability was assessed by the 3-(4,5-dimethylthiazol-2-yl)-2,5- diphenyltetrazolium bromide (MTT) assay as previously described [6]. The following four methods were used to assess drug-induced apoptotic cell death: detection of DNA fragmentation with the Cell Death Detection ELISA kit (Roche Diagnostics); western blot analysis for caspase activation and PARP cleavage; nuclear staining with Hoechst 33342;Annexin V analysis by flow cytometry. The cell death detection ELISA was performed to quantify the apoptotic index by detecting the histone-associated DNA fragments (mono- and oligo-nucleosomes) generated by the apoptotic cells [24]. In brief, the ovarian cells were treated with agents and were collected to prepare the cytosol fractions that contained the smaller fragments of DNA. Equal volumes of these cytosolic fractions were incubated in anti-histone antibody-coated wells (96-well plates), and the histones of the DNA fragments were allowed to bind to the anti-histone antibodies. The peroxidase-labeled mouse monoclonal DNA antibodies were used to localize and detect the bound fragmented DNA using photometric detection with 2, 29-azino-di-(3-ethylbenzathiazoline sulfonate) as the substrate, according to the manufacturer's instructions.

### Cell Fractionation

Cells were fractionated by differential centrifugation as previously described [54]. Briefly, cells were harvested and resuspended in three volumes of hypotonic buffer (210 mM sucrose, 70 mM mannitol, 10 mM Hepes, pH 7.4, 1 mM EDTA) containing 1 mM phenylmethylsulfonyl fluoride, 50 mg/ml trypsin inhibitor, 10 mg/ml leupeptin, 5 mg/ml aprotinin, and 10 mg/ml pepstatin. After gentle homogenization with a Dounce homogenizer, the cell lysates were centrifuged at 1,000×g for 5 min to remove unbroken cells and nuclei. The supernatant was collected and centrifuged at 10,000×g to pellet the mitochondria-enriched heavy membrane fraction. The supernatant was further centrifuged at 100,000×g to obtain a cytosolic fraction.

### SDS-PAGE and Immunoblotting

SDS-PAGE and immunoblotting were performed as described elsewhere [54]. Briefly, the cells or the membrane fractions were resuspended in a lysis buffer containing Nonidet P-40 (10 mM Hepes, pH 7.4, 2 mM EGTA, 0.5% Nonidet P-40, 1 mM NaF, 1 mM NaVO4, 1 mM phenylmethylsulfonyl fluoride, 1 mM dithiothreitol, 50 µg/ml trypsin inhibitor, 10 µg/ml aprotinin, and leupeptin) and were placed on ice for 30 min. The lysates were centrifuged at 12,000 ×g for 12 min at 4 °C, and the protein concentration was measured. Equivalent samples (30 µg of protein) were subjected to SDS-PAGE on 12% gels. The proteins were then transferred onto nitrocellulose membranes and probed with the indicated antibodies followed by the appropriate secondary antibodies conjugated to horseradish peroxidase (KPL, Gaithersburg, MD). Immunoreactive bands were visualized using enhanced chemiluminescence (Pierce). The molecular sizes of the proteins detected were determined by comparison with prestained protein markers (Invitrogen).

### Phospho-Amino Acid Analysis

Ovarian cancer cells were metabolically labeled with [^32^P] orthophosphoric acid for 120 min and treated with ATO for 48 h. ^32^P-Labeled BIM was immunoprecipitated using an agarose-conjugated BIM antibody, separated by SDS-PAGE and transferred to a polyvinylidene difluoride membrane. The membrane slice containing BIM was excised and hydrolyzed in 5.7 N HCL (Pierce) at 110 °C for 90 min under a vacuum. Cold phosphoserine, phosphothreonine and phosphotyrosine were added to the sample. Next, they were applied together onto a TLC plate and separated by electrophoresis utilizing a Multiphor II apparatus (Amersham Biosciences) with an equal volume of pH 1.9 and pH 3.5 buffers as described previously [55]. The location of phospho-amino acids was determined by ninhydrin staining and autoradiography.

### Determination of PP2A activity

A nonradioactive, malachite green-based serine/threonine phosphatase assay kit (Upstate Biotechnology, Lake Placid, NY, USA) was used to measure phosphatase activity [39]. Briefly, 5 µg of cellular proteins extracted in lysis buffer were incubated with 175 µM of the phosphopeptide (KIpTIRR) and the PP2A buffer (20 mM MOPS, pH7.5, 60 mM 2-ME, 0.1 M NaCl and 0.1 mg/ml serum albumin) in a total volume of 25 µl. Reactions were started by the addition of the phosphopeptide substrate and conducted for 10 min at room temperature. The reactions were terminated by the addition of malachite green solution, and the solution was left for 15 min to allow color development. Next, the plate was read at 650 nm with a microplate reader, and the amount of released phosphate was calculated from a standard curve. Although the reaction buffer and phosphorylated substrate in this assay were designed to detect PP2A, this assay may not fully discriminate between PP2A and PP1. Therefore, the sensitivity of the measured phosphatase activity to 0.2 µM okadaic acid (OA), which inhibits PP2A but not PP1, was determined for the assay.
